# Ideal time to replace isotonic crystalloid intravenous fluids and sets

**DOI:** 10.18849/ve.v10i3.47

**Published:** 2025-09-22

**Authors:** Erik Fausak+

**Keywords:** HANG TIME, IV FLUIDS, IV SETS, REPLACEMENT FLUIDS, SMALL ANIMAL

## Abstract

**Knowledge Summary Update:**

This paper is an update to 'Can I Hang? Ideal Time to Replace Isotonic Crystalloid Intravenous Fluids and Sets to Prevent Fluid Contamination and Blood Stream Infection: a Knowledge Summary' by Fausak et al. (2016).

Please click the link to view the original paper: https://doi.org/10.18849/ve.v1i4.47 .

**PICO question:**

In dogs and cats does the changing of IV fluids every 96 hours, compared to longer durations, reduce the risk of contamination in the bag?

**Clinical bottom line:**

**Category of research:**

Incidence.

**Number and type of study designs reviewed:**

Two prospective studies and a Cochrane systematic review for human patients.

**Strength of evidence:**

Weak.

**Outcomes reported:**

There was some consistency between the human systematic review and clinical experimental design in the veterinary intensive care unit (ICU). Another experimental study created some heterogeneity in results, where fluids were not contaminated for a long time (60 days), but two factors limit this study’s external validity, it was conducted in a clean laboratory environment and ports were wiped with alcohol before culturing samples. Both veterinary studies are limited as they only explored intravenous (IV) fluid bags which had no additives and were not connected to live animals or IV sets.

**Conclusion:**

Based on two prospective veterinary experiments and one human Systematic Review (meta-analysis), fluid bags and IV sets should be changed every 72–96 hours. Additionally, supportive evidence suggests that environmental cleanliness and creating a routine of wiping ports with alcohol prior to injection or withdrawal may significantly decrease the likelihood of fluid contamination. This certainly seems to be an area that needs more experimental studies with a low risk of bias on clinical patients.

## How to apply this evidence in practice

The application of evidence into practice should take into account multiple factors, not limited to: individual clinical expertise, patient’s circumstances and owners’ values, country, location or clinic where you work, the individual case in front of you, the availability of therapies and resources.

Knowledge Summaries are a resource to help reinforce or inform decision-making. They do not override the responsibility or judgement of the practitioner to do what is best for the animal in their care.

## Clinical scenario

Your practice currently keeps patients on fluids until they run out, you as a veterinary technician, are concerned that the bag may become contaminated and put the patient at risk, you would like to identify the ideal time to replace fluid bags connected to a patient by digging into the existing evidence. Additionally, as the same IV bags are used for different patients for subcutaneous and surgical fluid rate delivery, is there a concern for how long those bags are used to prevent cross-contamination?

## The evidence

Results included two prospective studies (not blinded) (Guillamin et al., 2017; Mathews & Taylor, 2011) and a Cochrane meta-analytic systematic review for human patients (Ullman et al., 2011). Due to limitations of all studies included with high risk of bias and not including veterinary patients create a weak base of evidence.

## Summary of the evidence

**Guillaumin et al. (2017):** Influence of hang time and location on bacterial contamination of intravenous bags in a veterinary emergency and critical care setting **Aim: **To determine, in a realistic clinical environment, rates of contamination to IV bags in a ICU and emergency room setting.

Population:	Fluid bags – Lactated Ringers Solution (LRS).
Sample size:	90 intravenous (IV) 1 litre fluid bags of lactated balanced-electrolytes solution.
Intervention details:	LRS intravenous bags were placed in an emergency room and intensive care unit of a busy academic hospital. Group 1 (n = 30) in Emergency Room (ER) above sharps container used for patient stabilisation. Group 2 (n = 30) in ER above sink and used to serve two tables for minor surgical procedures. Group 3 (n = 30) in Intensive Care Unit ( ICU )above hose and sink. All bags were punctured three times daily with an 18G needle attached to a 1–3 ml syringe (0800, 1200, and 1800 hours) and hung in the hospital's ICU and ER environment to simulate clinical usage. Aseptic technique was not performed when withdrawing fluids from the fluid bags (like wearing sterile gloves or swabbing ports with alcohol). Fluid sampling and port swabbing for bacteriological analysis occurred on days 0, 2, 4, 7, and 10. Port contamination: On days 0, 2 4, 7 and 10, culturette was rolled across the port surface and inoculated on a blood agar plate, streaked with an inoculation loop. Colonial growth was enumerated and phenotypically described – no data for day 10 was used, it was only used as a data point for day 7. Fluid contamination: On days 0, 2, 4, 7 and 10, 50 mls were withdrawn in a 60 ml syringe with an 18G needle twice each sample time and filtered through a 0.45 µM filter and collect the bacterial load via vacuum manifold, then the filter sheet was placed on Columbia agar with 5% sheep blood. Contamination of a bag was defined by the following conditions on discovery of bacteria (of the same phenotype): Presence of at least 1 bacterial colony. Increase of bacterial counts across 2 consecutive sample times.
Study design:	Prospective trial (non-randomised, non-blinded).
Outcome Studied:	Fluids and ports were cultured for colonisation of bacteria from IV bags at each location: ICU, ER over sink, and ER above sharps. Presence and increase of bacteria of the same phenotype were conditions for contamination.
Main Findings (relevant to PICO question):	No fluids from the bags in the ICU had bacterial contamination during the course of the study. In the ER, bags over the bin were at 1.1% contamination and 1.7% over the sink by day 4 and reached a maximum fluid contamination of 6.7% by day 7. All fluid ports were eventually contaminated. Port colonisation of bacteria occurring on day 0 was 0% in the ICU, and 3.3% over the bins and 6.7% over the sink. Day 2 was 3.3% in ICU, 6.6% over bins, and 23.3% in ER over sink. By day 4, ICU had 6.6% ER bins at 13.3%, and 33% over ER sink, day 7 ended with 33.3% contamination in ICU, 16.7% over ER bins, and 43% over ER-sink. Higher contamination rates occurred with bags above the sink than above sharps containers (bins). ICU (the cleanest environment) had overall lower rates of contamination of fluids and ports until port contamination over the ICU sink exceeded contamination over ER bins (sharp containers) on day 4. 98.2% compliance in study protocol. Combined bacterial growth on ports reached 8.1 CFU/port by day 10 with 95% confidence interval (0.005–16.2).
Limitations:	Daily checks might have been preferable since contamination occurred between days 2 and 4. The study tried to emulate clinical environment, but the authors (Guillaumin et al) concede that more frequent bag punctures should occur. Authors acknowledge this study was not addressing bacteremia and fluid bags – patient illness vs. growth of bacteria. This was essentially in vitro, where no patient was directly impacted or connected to fluids.

**Matthews & Taylor (2011):** Assessment of Sterility in Fluid Bags Maintained for Chronic Use **Aim: **To determine, in a laboratory environment, rates of contamination to IV bags.

Population:	Lactated Ringers Solution (LRS) bags used for subcutaneous delivery.
Sample size:	29 LRS bags.
Intervention details:	This study analysed intravenous (IV) Fluid bags and had two groups: Control: bags were removed from their plastic covering and a 1 ml sample was collected from fluid bag and interior bag wall was cultured. They were sampled again at 30 and 60 days without any needle penetration and fluid withdrawal. Intervention: the bag was punctured by a 3 ml syringe and 22G needle on a daily basis. Culture of injection port was collected after being wiped with alcohol and 1 ml was withdrawn on days 0, 7, 14, 21, 30, and 60.
Study design:	Randomised controlled non-blinded trial.
Outcome Studied:	Bacterial culture of fluid from aseptic technique of withdrawing fluid (wiping ports with alcohol before sampling and using sterile needle and syringe).
Main Findings (relevant to PICO question):	Day 60 resulted in bacterial growth of *Acinetobacter lwoffi* and *Staphylococcus* spp in two bags. No bags were contaminated before 60 days, and bags that were contaminated were in the injectable group, not the control group.
Limitations:	Methodology seems different from the previous two studies with alcohol prep of bag prior to culturing. This can confound study comparison. Longer gap between sampling dates, like day 0 and day 7. Essentially in vitro as bags were not on a set or delivered to a patient directly.

**Ullman et al. (2013):** Optimal timing for intravascular administration set replacement **Aim: **To examine the large body of human evidence to determine frequency fluids and IV Sets should be replaced.

Population:	Adult and neonatal human patients on central or peripheral intravenous (IV) and arterial lines with fluids being delivered over a period of time.
Sample size:	5001 human patients (16 studies).
Intervention details:	Human adult and neonatal patients receiving fluid therapy had their fluid lines evaluated for contamination at varying frequencies.
Study design:	Systematic Review.
Outcome Studied:	IV fluid colonisation and blood stream infections of patients on IV fluids by meta-analysis.
Main Findings (relevant to PICO question):	IV fluids and sets should be replaced every 96 hours unless containing blood products or parenteral nutrition. Neonates may warrant special consideration and more frequent IV set changes, based on two studies that were providing parenteral nutrition. More frequent IV set changes than 96 hours did not show any signs of increased infection: Relative Risk 1.08 with 95% confidence interval 0.7 to 1.86**.**
Limitations:	All studies included were not blinded and had a high risk of bias; they all received low quality scores. This systematic review did not investigate or address IV bag or set replacements greater than 96 hours.

## Appraisal, application and reflection

The updated search from the original 2016 Knowledge Summary was conducted similarly with some exceptions (Fausak et al*.*, 2016). Google Scholar was listed as a place searched in the original paper, but without clear indication of saturation (threshold) in results. Even limiting by language and years (2015 to current) resulted in 16,000 studies which would be very difficult to look at every title and abstract. Due to this large return of literature that was not relevant or reproducible, Google Scholar was dropped from our formal list of search engines on the update. The updated search was from 2015 to 2024 and we changed the language filter to English only instead of English and French. Additionally, PubMed created a systematic review filter since the original Knowledge Summary. Two searches were done in PubMed, one with the Systematic Review and Guideline filter and the other looking at just animal studies (Other Animal filter). A conference abstract featured in the original Knowledge Summary (Guillamin et al., 2013) was later published as a full study (Guillamin et al., 2017), allowing the author of this updated Knowledge Summary to undertake a full appraisal of the evidence.

Recommendations from the updated search do not really change from the original bottom line. In a veterinary clinical environment, changing intravenous (IV) sets and fluids between 72 and 96 hours have some support, per comparative guidance for human patients from the U.S. Centers for Disease Control and Prevention (CDC., 2011). Guillamin et al. (2017) suggests that veterinary contamination occurs more frequently than human, partially because of fluid type (Lactated Ringers Solution (LRS) versus 0.9% Sodium Chloride (NaCL)). Guillamin et al. (2017) also found higher risk of contamination with aerosolisation from sink activities and that bags probably should not be hung above sinks. The approach of Guillamin et al. (2017) had no measures to reduce contamination (wearing exam gloves or using alcohol to wipe ports). Guillamin et al. (2017) also felt that due to the veterinary environment and fluid type, it may closer match human paediatric recommendations of more frequent changes (closer to 72 hours). It is important to note that this Knowledge Summary does not address ideal time to replace or change IV catheters.

Fluid contamination that can lead to blood stream infections appear to be a fairly low risk to patients in human medicine (Ullman et al., 2013). In active and less clean environments, contamination of fluids seem to occur within four days of use (Guillaumin et al., 2013; Ullman et al., 2013). One well-designed study found that even with multiple patients, fluids were not contaminated in 60 days, but the sampling site was wiped with alcohol which may have affected the culture sensitivity, and since the environment was experimental, the facilities may have been much cleaner than a typical veterinary environment (Matthews & Taylor, 2011). One consistent theme the evidence suggests is that fluid and port contamination is directly related to the cleanliness of the surrounding environment.

The bottom line is that most IV fluids can ideally be changed every 72–96 hours with low risk of blood stream infection, but the evidence-base to support it remains very poor. While contamination may occur within 72 hours according to Guillamin et al. (2017), this is not based on a culture on day three, but on the contamination of fluids on day four. Percent contamination in Guillaumin et al (2017) in the veterinary clinical environment seems to be similar to the human meta-analysis (Ullman et al., 2013). Due to different study designs, it is hard to say where Matthews & Taylor (2011) fits in this spectrum since fluid contamination was not reached for 60 days. This could be due to Matthews & Taylor (2011) having a much smaller sample size in a much cleaner laboratory environment with more astringent methods being employed (for instance, wiping ports with alcohol).

Sabino & Weese (2006) examined factors for multi-dose vial contamination in veterinary practice. Based on two prospective control studies published in the article, vial top contamination is one of the largest factors for contaminated vial fluid. Swabbing the vial top resulted in a decline of 42% vial fluid contamination to 0% vial fluid contamination, much like Matthews & Taylor (2011) showing no fluid contamination when wiping ports with alcohol. As Sabino & Weese (2006) were not examining fluid bags, the study was not included for consideration in the PICO. Guillamin et al. (2017) found a 17.8% bacterial contamination of ports by day four; a likely source to introduce fluid contamination. It is important to know that both veterinary studies did not inject any additive into the bags and only withdrew volume. It is also important to note that the two veterinary studies were in no way used on patients which could introduce another important variable for contamination.

Future research that examines the cleanliness of personnel handling fluids and contamination of the fluids might be a very important avenue of examination. None of the included veterinary studies had animals attached to IV fluids. Also, none of the veterinary studies examined additives to bags like KCl, other drugs or vitamins., including from multi-dose versus single-dose vials. Additionally, more approaches that examine the multiple ways fluids are used in the veterinary environment should be examined. In extremely clean and aseptic surgical suites, is using the same bag between patients any cause for concern? Or are subcutaneous fluids used across multiple patients any more risky, particularly in a general practice or emergency room setting?

## Methodology

**Search Strategy:** 

Databases searched and dates covered:	CAB Abstracts on VetMed Resource 2015 to 3 December 2024 Medline on PubMed Platform 2015 to 3 December 2024
Search strategy:	VetMed Resource: (Intravenous AND set AND replacement) OR (intravenous AND fluid AND bag AND contamination) OR (“fluid therapy” AND contamination) English filter yr:[2015 TO 2024] 1 AND 2 AND 3 PubMed (search and translation): Human literature: Search: (Intravenous AND set AND replacement) OR (intravenous AND fluid AND bag AND contamination) OR ("fluid therapy" AND contamination) Filters: Guideline, Systematic Review, from 2015–2023 Animal Search in PubMed: Search: (Intravenous AND set AND replacement) OR (intravenous AND fluid AND bag AND contamination) OR ("fluid therapy" AND contamination) Filters: Other Animals, from 2015–2023
Dates searches performed:	3 December 2024

**Exclusion / Inclusion Criteria:** 

Exclusion:	Parenteral nutrition, human primary studies, and narrative reviews.
Inclusion:	IV Fluid administration set changing, only systematic reviews for human participant studies, and English-language publications.

**Search Outcome:** 

Database	Number of results	Excluded – not related to IV set replacement	Excluded – author's previous Knowledge Summary	Excluded – not in English	Total relevant papers
VetMed Resource	23	19	1	2	1
PubMed (systematic review and guideline filter)	2	2	0	0	0
PubMed (other animal filter)	12	11	0	0	1
Total relevant papers when duplicates removed					1

## Acknowledgements

The original work done by my co-authors on the first draft: Samantha Rae Spelts and Rebecca Lee Brown.

## ORCiD

Erik Fausak: https://orcid.org/0000-0002-0510-0153

### Conflict of Interest

The author declares no conflicts of interest.
